# Revisiting the association of sedentary behavior and physical activity with all-cause mortality using a compositional approach: the Women's Health Study

**DOI:** 10.1186/s12966-021-01173-0

**Published:** 2021-08-10

**Authors:** Jairo H. Migueles, I-Min Lee, Cristina Cadenas Sanchez, Francisco B. Ortega, Julie E. Buring, Eric J. Shiroma

**Affiliations:** 1 Department of Physical and Sports Education, Faculty of Sport Sciences, PROFITH “PROmoting FITness and Health through physical activity” Research Group, Sport and Health University Research Institute (iMUDS), University of Granada, Granada, Spain; 2 Division of Preventive Medicine, Brigham and Women’s Hospital, Harvard Medical School, Boston, MA USA; 3 Department of Epidemiology, Harvard T.H. Chan School of Public Health, Boston, MA USA; 4 Institute for Innovation and Sustainable Development in the Food Chain (IS-FOOD), Public University of Navarra, Pamplona, Spain; 5 Laboratory of Epidemiology and Population Science, National Institute on Aging, 251 Bayview Blvd, Baltimore, MD 21224 USA

## Abstract

**Background:**

While physical activity has consistently been associated with decreased mortality rates, it remains unknown if there is a single “ideal” combination of time in physical activities of different intensities and sedentary behavior (SB) associated with the lowest rate. This study examined the associations of combinations of time in moderate-to-vigorous intensity (MVPA), higher-light intensity (HLPA), lower-light intensity activities (LLPA), and SB with mortality rates in older women.

**Methods:**

This prospective cohort study included 16,676 older women from throughout the United States enrolled in the Women’s Health Study. Women wore accelerometers on their hip from 2011 to 2015 and were followed through 2017 (mean (SD) of 4.3 (1.1) years). Deaths were confirmed with medical records, death certificates, or the National Death Index. Compositional Cox regression models were used.

**Results:**

The mean (SD) age was 72 (5.7) years at accelerometer wear; 503 women died. Compared to the least active women (mean, 3 min/day MVPA, 27 min/day HLPA, 162 min/day LLPA, and 701 min/day SB): compositional models showed an inverse L-shaped dose-response association of MVPA replacing other behaviors with mortality rates mortality rates (*P* = .02); SB relative to LLPA, HLPA, and MVPA was directly associated with mortality rates in a curvilinear dose-response manner (*P* < .001); replacing 10 min of SB for MVPA (HR (95% CI) = .86 (.73–.98)) or for HLPA (HR (95% CI.94 (.88–1.00)) associated with 14 and 6% lower mortality rates, respectively; a 47% risk reduction (HR [95% CI] = .53 [.42–.64]) was observed among women meeting physical activity guidelines (mean, 36 min/day MVPA, 79 min/day HLPA, 227 min/day LLPA and 549 min/day SB); and similar mortality rate reductions of 43% (HR (95% CI) = .57 (.41–.73)) were observed with increases in HLPA and LLPA without increasing MVPA, e.g., reallocating SB to 90 min/day of HLPA plus 120 min/day of LLPA.

**Conclusions:**

There was no “ideal” combination of physical activities of different intensities and SB associated with the lowest mortality rates. Of particular relevance to older women, replacing SB with light intensity activity was associated with lower mortality rates, and “mixing and matching” times in different intensities yielded equivalent mortality risk reductions.

**Supplementary Information:**

The online version contains supplementary material available at 10.1186/s12966-021-01173-0.

## Introduction

Physical inactivity has been shown to be associated with higher mortality rates, and this behavior increases with age [1]. While studies of physical activity have largely tended to focus only on moderate-to-vigorous intensity activity (MVPA), older adults are more likely to engage in light intensity activities (LPA) and sedentary behaviors (SB). Recent studies have shown that LPA and SB are independently associated with health [2, 3]. Physical activity guidelines recommend 150 min per week of MVPA, but there are currently no specific (quantitative, threshold-based) guidelines for LPA or SB [4, 5].

There has been a shift in research paradigm from investigating the associations of particular intensities of physical activity [3] or SB [6] (in isolation) to investigating the composition of the entire day [7, 8]. This paradigm is more “real life”, as the time in a day is finite (thus, doing more of one activity must mean a commensurate reduction in other behaviors). Further, investigating the composition of the day spent on the different behaviors allows researchers to describe and compare outcomes associated with the different combinations of MVPA, LPA and SB [8, 9].

However, analysis focusing on the composition of the entire day is challenged by both physical activity assessment and statistical challenges. Self-reported behavior relies on recall and may have less detail as questionnaires tend to categorize time spent into ranges of time. Further, LPA are not well reported on questionnaires and tends to assume homogeneity of these activities (i.e., do not differentiate between HLPA and LLPA). Accelerometry, which is an objective and continuous measure of physical activity and SB, can remove many of these challenges. The continuous recording of movement allows for a more precise volume estimate across the entire spectrum of physical activity intensities. This may be particularly relevant for older adults, who primarily tend to engage in activities of HLPA and LLPA [3]. With regard to statistical challenges, accounting for a continuous range of time spent in multiple behaviors while acknowledging that the day must always total 24-h requires specialized statistical methods (an increase in one category requires a decrease in another) [10, 11]. This can be addressed using compositional data analysis, which has been recently applied to the field of physical activity and health research [7, 8].

While previous studies have shown associations of physical activity, SB, and mortality, it remains unknown whether is there a single “ideal” combination of these behaviors associated with the lowest mortality rates. This study aims to compare mortality rates within various combinations of MVPA, HLPA, LLPA, and SB among older women using accelerometry and compositional data analysis.

## Methods

### Participants and setting

Women were drawn from the Women’s Health Study (WHS), a randomized controlled trial (1992–2004) of aspirin and vitamin E in the prevention of cancer and cardiovascular disease [12, 13]. Upon trial completion, women were invited to continue in an observational follow-up study (2004 – current). In 2011, an ancillary study examining accelerometer-assessed physical activity was launched [14, 15]. Women were asked to wear a triaxial accelerometer (ActiGraph GT3X+, ActiGraph Corp, Pensacola, FL, US) on the hip for 7 days during waking hours. Accelerometer data collection took place during 2011–2015. Of 17,466 women with any recorded accelerometer data, 16,741 (96%) had data from ≥10 h per day on ≥4 days, a standard criterion for valid wear [16]. For the present analyses, we included 16,676 women with data in all covariates (see below). This study was approved by the Institutional Review Board of Brigham and Women’s Hospital (Boston, MA) and women provided written consent to participate. This report followed the Strengthening the Reporting of Observational Studies in Epidemiology (STROBE) reporting guidelines for cohort studies.

### Accelerometer-assessed physical activity

Accelerometers are wearable devices that measure accelerations (i.e., movement) of the body segment to which the monitor is attached (in this case, hip). These devices are widely used in the physical activity epidemiology field to objectively record physical activity and SB patterns. Accelerometer data collection and processing criteria have been described in detail elsewhere [17]. Briefly, raw data were aggregated into counts per minute, a standard measure of activity intensity using ActiLife software (ActiGraph Corp, Pensacola, FL, US). Non-wear time was defined as 90 min of consecutive 0 counts per minute with an allowance of 1–2 min of 0–99 counts per minute surrounded by two 30-min windows of 0 counts per minute [18]. For the present study, we used previously published cut points for hip-worn devices using data from the vertical axis to categorize these behaviors: MVPA (≥ 1952 counts per minute), HLPA (761–1951 counts per minute), LLPA (101–760 counts per minute), and SB (≤ 100 counts per minute) [18, 19]. These cut points were defined upon the observation of a linear relationship between counts per minute and activity intensity represented by metabolic equivalents (r^2^ = 0.82) [19].

### Participant characteristics, health status and mortality

Women reported demographic characteristics, health habits and medical history on annual questionnaires. Information from the questionnaire closest to accelerometry assessment was obtained on age, smoking, alcohol intake, postmenopausal hormone use, general health status, cancer screening, parental history of myocardial infarction and family history of cancer. Additionally, dietary habits were assessed using a semiquantitative food questionnaire at the beginning of the WHS [20]. Reported diagnoses of cardiovascular disease and cancer were confirmed using medical records. Women were followed through December 31, 2017 for mortality, with deaths confirmed with medical records, death certificates, or the National Death Index.

### Statistical analysis

As women were asked to wear their accelerometers during time awake, a complete 24-h analysis was not possible. We thus used total waking hours to represent the day. The accelerometer-determined time in MVPA, HLPA, LLPA, and SB was calculated as proportions of the total wear time to remove the potential bias of women wearing the monitor for different amounts of time. The means of time spent in each behavior were calculated and transformed to proportions of wear time (i.e., each behavior is considered as a relative amount of time to the total wear time), providing the mean composition of the behavior during waking hours. Eighty women recorded zero-values in MVPA (i.e., no time spent in this category), which is problematic for compositional data analysis. As such, we imputed these zeroes with the log-ratio Expectation-Maximization algorithm proposed by Palarea et al. [21] using the zCompositions R package [22].

We used compositional Cox proportional hazard models [11] to study the associations of the different combinations of behaviors or time-use composition (i.e., in MVPA, HLPA, LLPA, and SB) with all-cause mortality, adjusting for the necessity of the total of the different behaviors to sum to the full day. This regression method allows not only examination of different profiles (i.e., groups of women with varying combinations of time spent in the difference behaviors), but also combinations of activity intensities on a continuous scale. The model predictions represent the hazard ratio (HR) associated with a given composition, compared to a referent composition. For our referent composition, we used the compositional mean of the women in the first quartile of total activity (mean, 3 min in MVPA, 27 min in HLPA, 162 min in LLPA and 701 min in SB per day). Tests based on Schoenfeld residuals were used to check the proportional hazards assumptions [23]. No violations were observed. Martingale residuals against each physical activity intensity and SB data (without compositional transformation) showed that the dose-response was likely to be non-linear. Models were adjusted for age, smoking, alcohol intake, saturated fat intake, fiber intake, fruit and vegetables intake, postmenopausal hormone therapy, parental history of myocardial infarction, family history of cancer, general health, history of cardiovascular disease, history of cancer and cancer screening.

Four main analyses examining different combinations of behaviors were performed. First, we calculated the mortality rate associated with increasing time spent in a single behavior (on a continuous time scale) while proportionally reducing time spent on the other behaviors (using the proportions in the referent time-use category of the least active quartile). Second, we analyzed the associations with pair-wise reallocations of time between behaviors (e.g., moving 10 min per day of SB to 10 min per day of MVPA) while keeping the other two behaviors constant (in this case, time in HLPA and LLPA remain unchanged). To compare the compositional data models with the more frequently used survival analysis technique, we performed (standard) Cox proportional hazard models to calculate the mortality hazard ratio for reallocating 30 min/day from SB to each physical activity intensity. For such purpose, we included all behaviors but one in the model, so that the model estimates represent the effect of increasing a behavior at the expense of the missing behavior. Third, we defined several specific time-use compositions: (a) displacing time from SB to MVPA to meet the physical activity guidelines [5]^;^ (b) displacing time from SB to LLPA and (c) SB to HLPA to approximate the HR found in (a); (d) the mean time-use composition of women in this study who met the physical activity guidelines; and (e) displacing time from SB to a combination of HLPA and LLPA to approximate the HR found in (d). All these specific time-use compositions were compared to the least active quartile. Finally, we used ternary plots (i.e., three-axis scatter plots) to estimate the HRs associated with time reallocations among three of the behaviors (e.g., SB, HLPA and MVPA) while keeping the fourth behavior constant (in this case, LLPA).

Sensitivity analyses were conducted by excluding women with cardiovascular disease or cancer at the time of accelerometer wear or follow-up shorter than 2 years. All analyses were performed in R v.4.0.0 (https://cran.r-project.org/) with open-source code for compositional data analysis (codes available at www.opencoda.net). Statistical tests were all 2 sided, with the level of significance set to .05.

## Results

A total of 503 of the 16,676 women died during a mean (SD) follow-up of 4.3 (1.1) years. The mean (SD) age of women was 72.0 (5.7) years (range, 62–101 years) at baseline in the ancillary accelerometer study (Table 1). Women wore their devices for a mean (SD) of 14.9 (1.3) hours per day, with 1.7% of time awake spent in MVPA, 7.1% in HLPA, 24.9% in LLPA and 66.3% in SB.

**Table 1:** Baseline characteristics of the women participating in this study

Characteristic	No. = 16,676 women
Age, mean (SD), y	72.0 (5.7)
Smokers, No. (%)	
Never	8404 (50.4)
Former	7688 (46.1)
Current	584 (3.5)
Alcohol use, No. (%)	
Never	6338 (38.0)
Rarely	1634 (9.8)
Monthly	6053 (36.3)
Daily	2651 (15.9)
Hormone therapy, No. (%)	
Never	11,995 (71.9)
Former	3027 (18.2)
Current	1654 (9.9)
General health, No. (%)	
Poor	4102 (24.6)
Fair/good	8355 (50.1)
Very good	3785 (22.7)
Excellent	434 (2.6)
Parental history of myocardial infarction, No. (%)	2401 (14.4)
Family history of cancer, No. (%)	4319 (25.9)
History of cardiovascular disease, No. (%)	400 (2.4)
History of cancer, No. (%)	1984 (11.9)
Cancer screening, No. (%)	13,674 (82.0)
Accelerometer wear time, mean (SD), min/d	891.4 (75.2)
Moderate-to-vigorous intensity activity, mean (SD), min/d	14.7 (16.5)
Higher-light intensity activity, mean (SD), min/d	63.6 (35.1)
Lower-light intensity activity, mean (SD), min/d	222.0 (52.9)
Sedentary behavior, mean (SD), min/d	591.3 (81.3)

### Increasing time in one behavior while proportionally reducing the rest

**Fig. 1:**
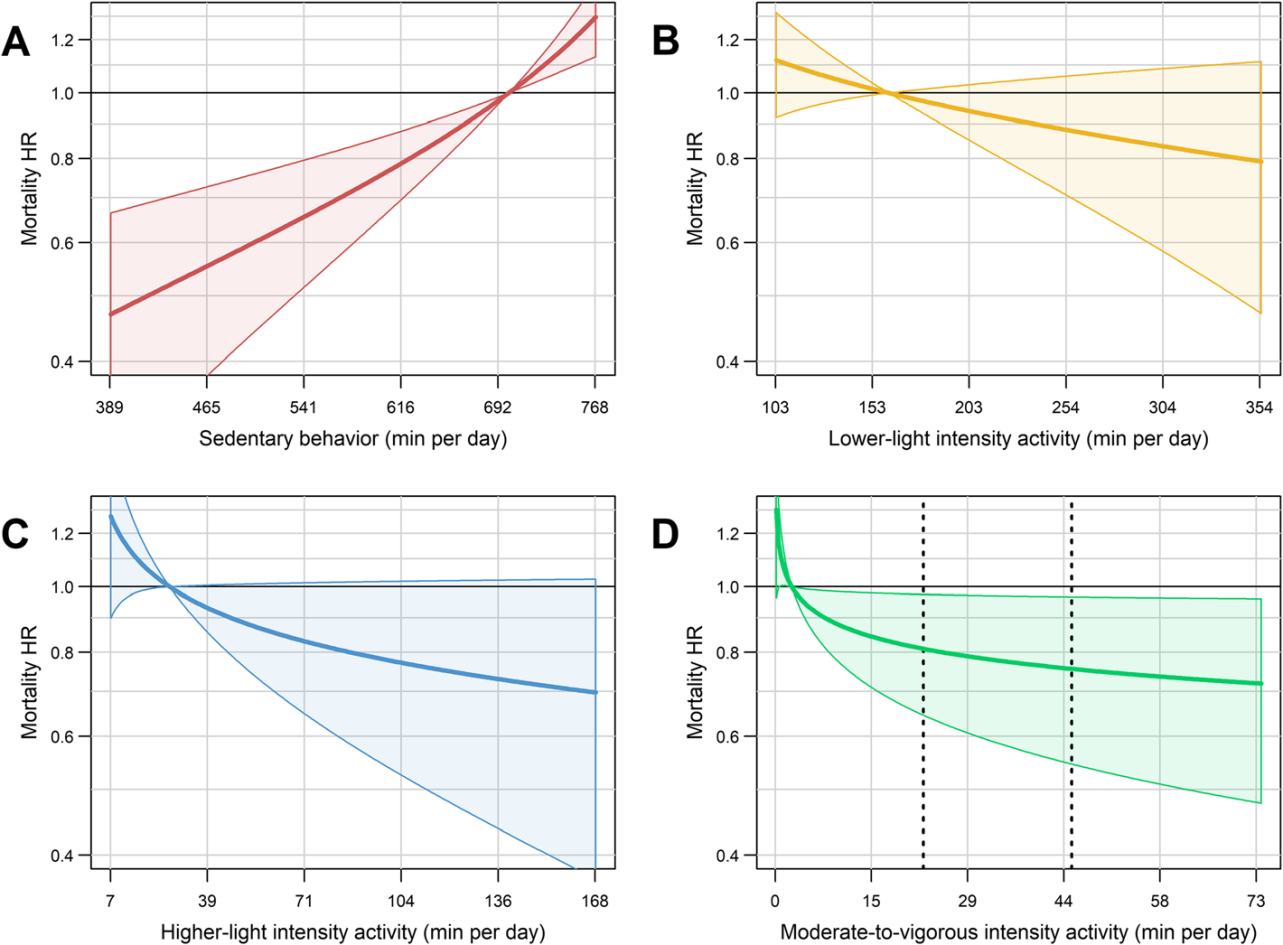
Dose-response associations of physical activity of different intensities and sedentary behavior with mortality **(**HRs are compared to the referent composition, the lowest quartile of total activity). Average awake wear time is 14.9 (SD = 1.3) hours per day Each line represents time in a behavior while proportionally^a^ reducing the others. Shaded areas represent the 95% confidence intervals. HR: hazard ratio. ^a^ Proportional to the referent composition, i.e., women in the lowest quartile of total activity: 3 min in moderate-to-vigorous, 27 min higher-light, 162 min lower-light intensity activity, and 701 min in sedentary behavior per day.

For all the analyses presented, the referent time-use composition is the average of the women in the least active quartile (mean, 3 min/day MVPA, 27 min/day HLPA, 162 min/day LLPA, and 701 min/day SB). More time in MVPA (with proportional decreases of time in SB, LPA) was associated with a reduced mortality rate (*P* = 0.02). We observed a dose-response association with a “L” shaped curve, with a stronger response among those with lower baseline activity levels (Fig. 1D). For example, increasing from 3 to 13 min per day of MVPA (10 min increase) was associated with a 14% lower mortality rate (HR [CI] = 0.86 [0.73 to 0.98]); however, increasing from 13 to 23 min per day (also 10 min increase) was associated with a 5% lower rate (HR [CI] = 0.81 [0.64 to 0.97]). Increasing HLPA while proportionately reducing the other behaviors showed a non-statistically significant association with lower mortality rates (*P* = 0.07). The dose-response relation was similar to that for MVPA, but with a lower magnitude of the HRs (Fig. 1C). For example, 27 to 37 min per day (10 min increase) increase in HLPA was associated with 6% lower mortality rate (HR [CI] = 0.94 [0.88 to 1.00]). More time in LLPA (with proportional reductions in the other behaviors) was not significantly associated with mortality rate (*P* = 0.18). Finally, SB (relative to physical activity of all intensities) was directly associated with mortality rate in a dose-response manner (*P* < 0.001) (Fig. 1A). Reducing SB from 701 to 641 min per day (60 min decrease) associated with a 18% lower mortality rate (HR [CI] = 0.82 [0.75 to 0.89]), while reducing from 641 to 581 min per day was (also 60 min decrease) associated with an additional 13% lower mortality rate, i.e., 31% lower than the least active women (HR [CI] = 0.69 [0.58 to 0.80]).

Sensitivity analyses excluding women with < 2 years of follow-up, or presenting cardiovascular disease or cancer at baseline were performed. Although in agreement, findings were attenuated in the sensitivity analysis, i.e., SB was significantly associated with higher mortality rate (*P* = 0.024, supplementary material, Fig. S1A); HLPA and MVPA showed similar dose-response curves as in the main analyses, but the associations were not significant (*P* > 0.126, supplementary material, Fig. S1A).

### Pair-wise reallocations of time between behaviors

**Fig. 2:**
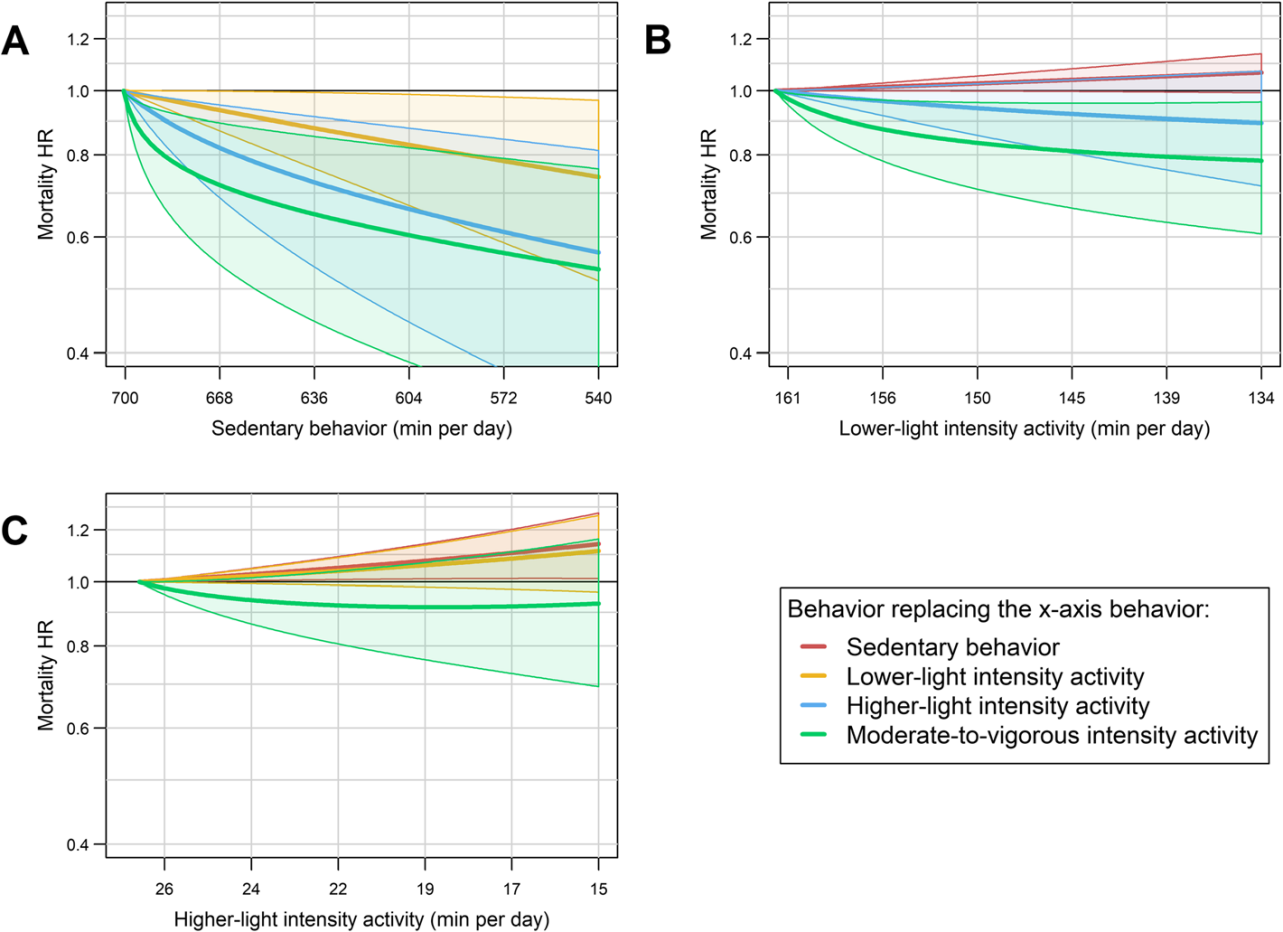
Mortality HRs for pairwise reallocations of time between behaviors while the remaining two behaviors are kept constant at the proportions in the referent composition (i.e., lowest quartile of total activity). Average awake wear time is 14.9 (SD = 1.3) hours per day. Shaded areas represent the 95% confidence intervals. HR: hazard ratio

**Fig. 3:**
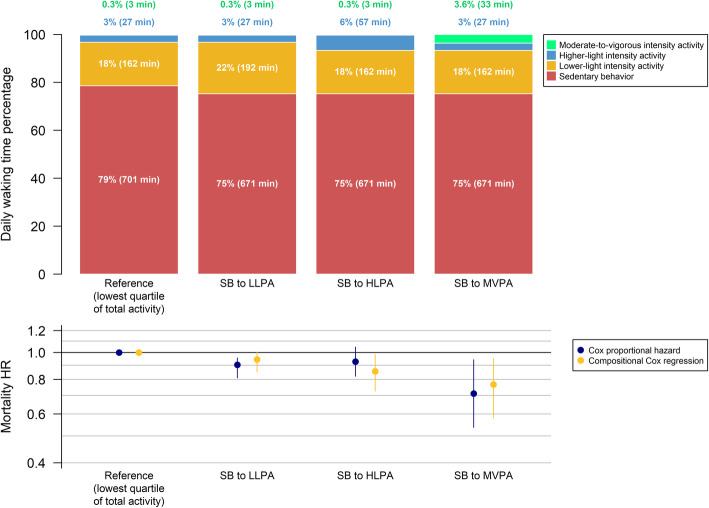
Specific time-use compositions arising from displacing 30 min/day from SB to each PA intensity and their estimated mortality HR and 95% confidence intervals (error bars) compared to the referent composition (i.e., lowest quartile of total activity) using standard and compositional Cox proportional hazard models. Average awake wear time is 14.9 (SD = 1.3) hours per day. HR: hazard ratio

Next, we investigated the difference in the mortality rate associated with pair-wise reallocations of time between behaviors (Fig. 2). Reallocating time from SB to either MVPA, HLPA or LLPA were all significantly associated with lower mortality rates (Fig. 2A). The magnitude of the association was larger as the intensity of the replacement behavior increased. For example, reallocating 20 min per day of SB to MVPA, HLPA or LLPA, was associated with 20% (HR [CI] = 0.80 [0.64 to 0.96]), 11% (0.89 [0.79 to 0.99]), and 4% (0.96 [0.92 to 1.00]) lower mortality rates, respectively. Reallocating time from LLPA to MVPA was associated with lower mortality rates (e.g., 20 min replacement: HR [CI] = 0.83 [0.67 to 1.00]) (Fig. 2B). Finally, displacing time from LLPA to HLPA (Fig. 2B), or from HLPA to MVPA (Fig. 2C) was not significantly associated with mortality rates. The mortality associations of reallocating 30 min/day from SB to each physical activity intensity is shown using both compositional survival and standard Cox survival models in Fig. 3. The associations were similar using both models; however, standard Cox proportional hazard models depict a more linear relationship while compositional models show an L-shaped curvilinear relationship as shown in Figs. 1 and 2.

### Pre-defined time-use compositions

**Fig. 4:**
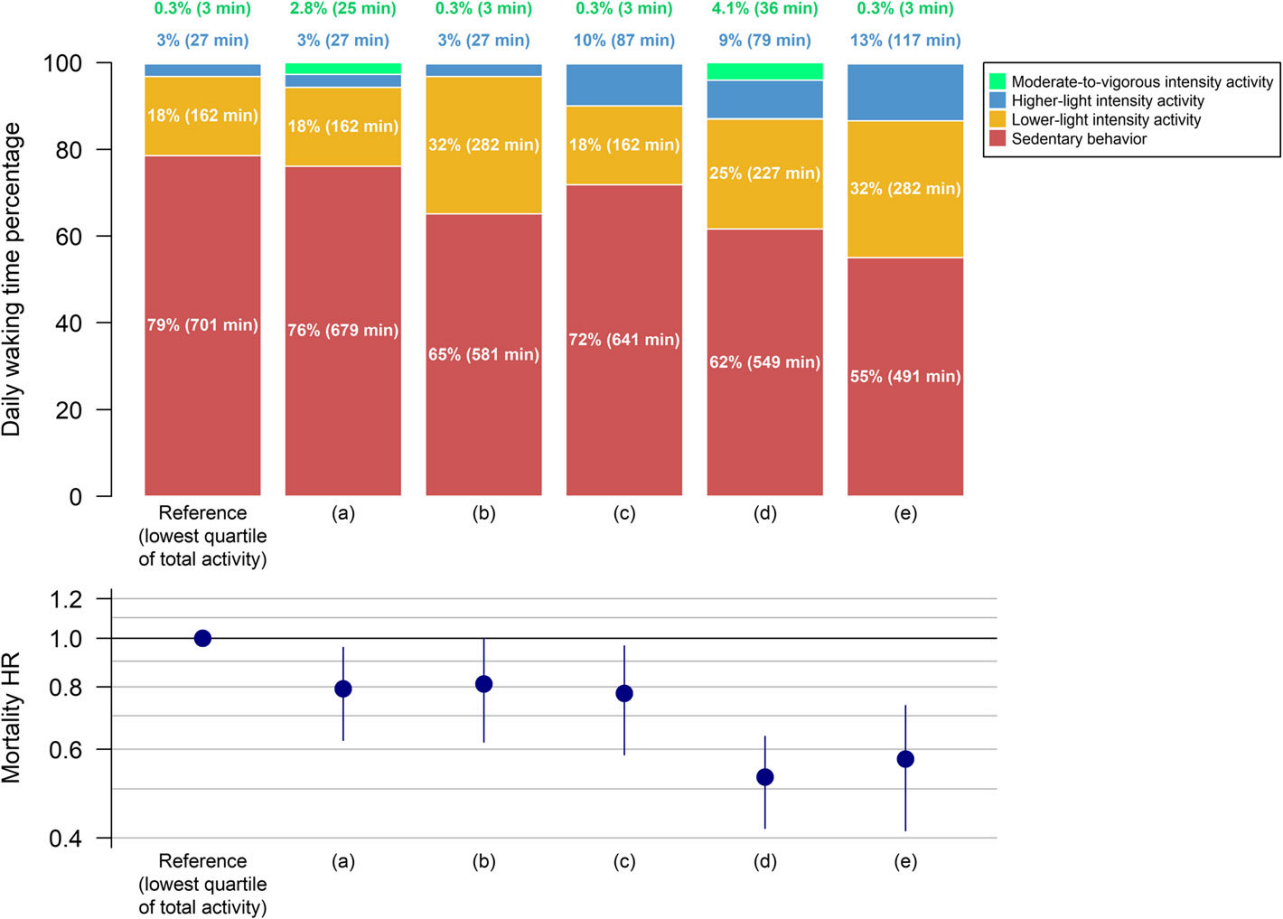
Specific time-use compositions and their mortality HR and 95% confidence intervals (error bars) compared to the referent composition (i.e., lowest quartile of total activity). Average awake wear time is 14.9 (SD = 1.3) hours per day. (a) Reallocation of 22 min per day from sedentary behavior to moderate-to-vigorous intensity activity (for a total of 25 min per day); all other behaviors remain the same. Twenty-two min per day of moderate-to-vigorous intensity activity approximately totals 150 min per week, which is recommended by current guidelines. (b) Reallocation of time from sedentary behavior to lower-light intensity activity to approximate the mortality HR found in (a): 120 min per day reallocated; all other behaviors remain the same. (c) Reallocation of time from sedentary behavior to higher-light intensity activity to approximate the mortality HR found in (a): 60 min per day reallocated; all other behaviors remain the same. (d) Mean time-use composition of women in this study who met the physical activity guidelines of 150 min per week of moderate-to-vigorous intensity activity (4149 women [25%]). (e) Reallocation of time from sedentary behavior to a combination of higher-light and lower-light intensity activity to approximate the mortality HR found in (d): 90 min to higher-light plus 120 min per day to lower-light intensity activity; moderate-to-vigorous intensity activity remains the same

Compared to the women in the lowest quartile of total activity, reallocating 21.5 min per day from SB to MVPA (i.e., 150 min per week, thereby meeting guidelines) was associated with 21% lower mortality risk (HR [95% CI] = 0.79 [0.63 to 0.96]). A similar risk reduction was obtained by reallocating 120 min per day from SB to LLPA (HR [95% CI] = 0.81 [0.62 to 1.01]) or 60 min per day from SB to HLPA (HR [95% CI] = 0.78 [0.59 to 0.97]). Women in this study who met physical activity guidelines had a 47% lower mortality rate (HR [95% CI] = 0.53 [0.42 to 0.64]) compared to those in the lowest quartile. This differs from the 21% risk reduction of changing SB to MVPA because these women also had more LPA compared to the lowest quartile. To approximate this 47% risk reduction without manipulating MVPA, this necessitated reallocation of 210 min per day of SB: 90 min to HLPA plus 120 min to LLPA (HR [95% CI] = 0.57 [0.41 to 0.73]) (Fig. 4**)**. This resulted in a composition of 3 min in MVPA, 117 min in HLPA, 282 min in LLPA and 491 min in SB per day.

### Ternary diagrams for the association of SB, LLPA, HLPA, and MVPA with mortality rate

**Fig. 5:**
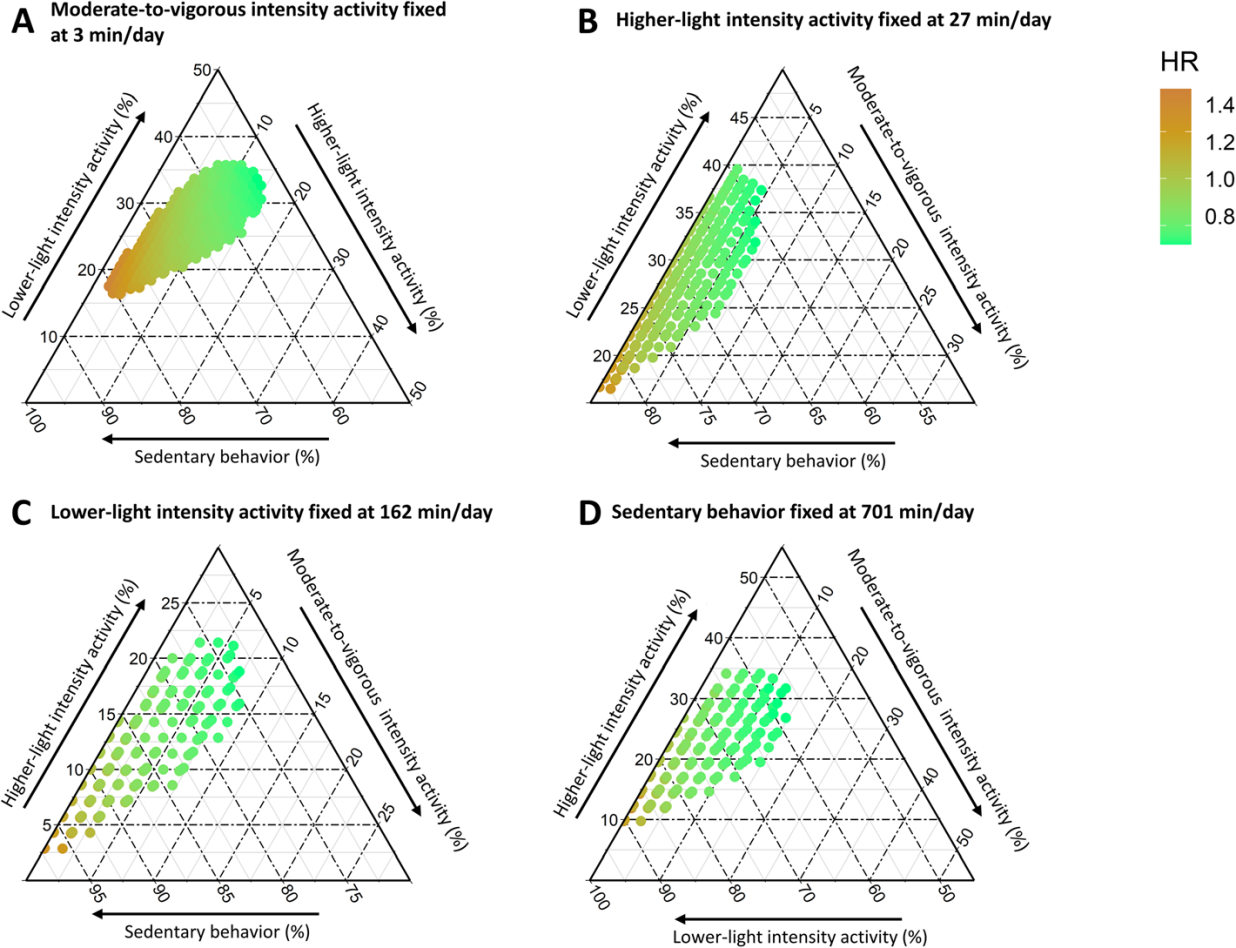
Ternary plots representing the predicted HR for combinations of the proportions of time spent in three behaviors while keeping the last behavior fixed at the proportion in the referent composition (the lowest quartile of total activity). Average awake wear time is 14.9 (SD = 1.3) hours per day. In panel **A**, the proportion of time in moderate-to-vigorous intensity activity is fixed; in panel **B**, the proportion of time in higher-light intensity activity is fixed; in panel **C**, the proportion of time in lower-light intensity activity is fixed; in panel D, the proportion of time in sedentary behavior is fixed. HR: hazard ratio

Ternary diagrams showing different compositions arising from time reallocations among three of the behaviors while keeping the fourth behavior constant (HRs are compared to the referent composition) are presented in Fig. 5. The heatmaps in the different ternary plots represent the mortality HR associated with every time combination on a continuous scale. As shown, similar predicted HRs (similar colors) were observed with many different compositions of behaviors.

## Discussion

To the best of our knowledge, this is the first study investigating the reallocations of time across MVPA, HLPA, LLPA and SB and their associations with mortality rates in older women. Presently, most public health physical activity guidelines focus only on MVPA. However, many recent guidelines are beginning to recognize potential health benefits from physical activity of any intensity, although there are no quantitative specifications for activities other than those of MVPA (e.g., “some physical activity is better than none”) [24–26]. This study demonstrates that other combinations of behaviors might provide similar benefits against mortality without focusing solely on MVPA as suggested by current guidelines. Furthermore, we observed that there was no single “ideal” combination of time spent in SB and physical activity of different intensities that was associated with the lowest mortality rates. By visually examining the ternary plots, it is evident that similar HRs can be obtained with different combinations of MVPA, LPA and SB.

The importance of LPA is especially relevant for those populations - such as older women - who have difficulty engaging in MVPA. The current guidelines of 150 to 300 min of MVPA may not be realistic for many older women. We observed reductions in mortality rates associated with LPA supporting that future guidelines should incorporate LPA, perhaps even both LLPA and HLPA. Examples of LLPA are washing or drying dishes; HLPA may include laundry, mopping, or walking at 1.5 mph among others [27, 28]. These represent feasible activities for older individuals. Similar to the current study, a recent harmonized meta-analysis in men and women found that 80 min (117 min in WHS) of HLPA or more than 5 h (282 min in WHS) of LLPA per day were needed to approximate the mortality rate observed among persons meeting the current physical activity recommendations [3]. However, the meta-analysis did not consider the time-use composition of all behaviors, so it is unknown how much LPA was performed in those with more MVPA, creating an uneven comparison as it examines only one behavior while multiple may differ. WHS found that those with higher MVPA also had higher LPA. The impact of this higher LPA should also be taken into account, for example, with compositional data analysis.

Consistent with previous studies [2, 3, 7, 8], we observed lower mortality rates as more time was reallocated from SB to physical activity of any intensity, supporting the ‘every move counts’ slogan recently promoted by the World Health Organization [29]. We also showed this holds true when changing multiple intensities at once with compositional analyses. In agreement, two previous studies using a similar approach observed that increasing MVPA at expenses of lower intensities was associated with lower mortality rates [7, 8]. Neither of these studies specifically investigated the relevance of LLPA and HLPA; thus, the time-use compositions are not directly comparable, and our findings should be considered novel in this regard. We found it relevant since part of the activity classified as LPA, what we refer to HLPA, may associate significantly with mortality rates. This observation is important from a public health perspective and it might have been ignored in previous studies. There is a previous study investigating specifically the relevance of LPA in women relative to cardiovascular disease incidence [2]. Although they did not use a compositional approach, they found that 1-h increase in LPA associated with 8% cardiovascular disease risk reduction [2], which is in line with, and extended by, our findings. Substitutions that resulted in reductions of MVPA when increasing LPA were not statistically significant even if SB is also decreased. However, while not statistically significant, the dose-response curves are similarly inversely associated with mortality as observed with MVPA. Otherwise, our predefined time-use composition showed that, theoretically, it is possible to obtain similar mortality risk reductions to the ones observed in women meeting the guidelines, without increasing the time in MVPA beyond the lowest activity quartile (i.e., 3 min per day). However, the amount of LPA needed to replicate the MVPA benefits may be unrealistically large (i.e., 210 min per day).

This study possesses several strengths. A major strength is the use of accelerometers which are able to capture detailed volume and intensity patterns of physical activity and SB. The study also included a large sample of older women from throughout the United States. The compositional data analyses mirror “real life” by considering differing time reallocations, on a continuous scale, across behaviors with the constraint that a day always has 24-h (as in the real world) – if one behavior is increased, the other(s) must commensurately decrease.

We observed that standard Cox proportional hazard models can also estimate time exchange between behaviors with similar hazard ratios, however, it is limited to increasing one behavior at a time. In addition, the linear dose-response of the standard model should be questioned. The associations with an increase MVPA from 0 min/day to 30 min/day and from 30 min/day to 60 min/day may be biologically different. Using standard Cox proportional hazard models, these scenarios are assumed to have the same HR, while compositional models provide the specific HR for each scenario (assuming a curvilinear dose-response). It remains unknown if using standard Cox proportional hazard models with a more complex model (such as quadratic terms) will change these results. Lastly, standard Cox proportional hazard analyses have not, for the most part, accounted for the constraint of the behaviors summing to a full day and the correlations associated with that.

### Limitations

This study did not have information on sleep, since women only wore the devices while awake. Thus, we are unable to make conclusions for a complete 24-h day. In light of this, we adjusted for different amounts of wear time between women. Hip-worn accelerometers may not accurately measure activities with a predominance of upper-body movements (e.g., dishwashing), detect body postures, or identify when carrying weight. Nevertheless, accelerometers are the best existent approach at the moment to objectively measure free-living activity with a low burden to the participants, particularly for activities of lower intensity. In this regard, we applied widely-used cut-points to classify physical activity intensities that were developed in younger adults than this study sample [19]. These absolute cut-points may misclassify actual MVPA for LPA in our sample, considering the age-related decrease in exercise capacity [30]. Research on the estimation of relative physical activity intensity or age- and sex-specific populational cut-points is needed to overcome the limitations of absolute cut-points. Furthermore, our time reallocation analyses are based on the observed time-use compositions across participants and not on actual within-participants changes in the time-use composition. Well-designed randomized controlled trials are needed to confirm that the benefits observed in the hypothetical reallocations of time actually take place. Physical activity guidelines to date have primarily been based on studies using self-reported physical activity; hence our findings using accelerometers to define “meeting physical activity guidelines” may not be directly comparable. Caution in the interpretation of the effect sizes of meeting the guidelines is advised. While we adjusted for many potential confounders, residual confounding cannot be entirely eliminated. Additionally, information on confounders was collected at a single point in time. Accelerometer-measured physical activity also was assessed at a single time point. However, in a sample of women, we found good consistency of accelerometer-measured physical activity over a period of 2–3 years (intraclass correlation ~ 0.7–0.8) [31]. Confounders were ascertained immediately prior and up to 3 years before the accelerometer data collection, except for the diet, which was ascertained at the start of the WHS trial (~ 2 decades before). Thus, the variability of the confounders along time can also influence our findings. Although we conducted sensitivity analyses, the reverse causation risk cannot be fully eliminated. To examine reverse causation, we conducted sensitivity analyses excluding women with cardiovascular disease or cancer, as well as those with a follow-up time of less than 2 years, showing similar results as the full sample. WHS is comprised of primarily white women of a higher socio-economic status, potentially limiting the generalizability of these findings.

## Conclusion

In a large cohort of older women, we found that no single combination of time in various physical activity intensities and SB was associated with the lowest mortality rates. Similar mortality rate reductions were observed with increases in LPA, without focusing solely on MVPA, as recommended by guidelines. As LPA may be more feasible for older individuals, this is important for informing future physical activity guidelines. The findings also suggest that older individuals can tailor their physical activity and SB patterns to match individual preferences with similarly associated mortality rates.

## Supplementary Information

**Additional file 1.**

## Data Availability

The datasets generated and/or analyzed during the current study are not publicly available but are available from the corresponding author on reasonable request.

## Abbreviations

WHS: Women’s Health Study
HR: Hazard ratio
CI: Confidence interval
MVPA: Moderate-to-vigorous intensity physical activity
LPA: Light intensity physical activity
LLPA: Lower-light intensity physical activity
HLPA: Higher-light intensity physical activity
SB: Sedentary behavior
