# Supplementary material for: Revisiting the association of sedentary behavior and physical activity with all-cause mortality using a compositional approach: the Women's Health Study
Source: Int J Behav Nutr Phys Act. 2021 Aug 10;18:104. doi: 10.1186/s12966-021-01173-0 (PMC8353824; doi:10.1186/s12966-021-01173-0)
Supplement: Supplementary file 1 — Additional file 1. [file 12966_2021_1173_MOESM1_ESM.docx]

# Supplementary material


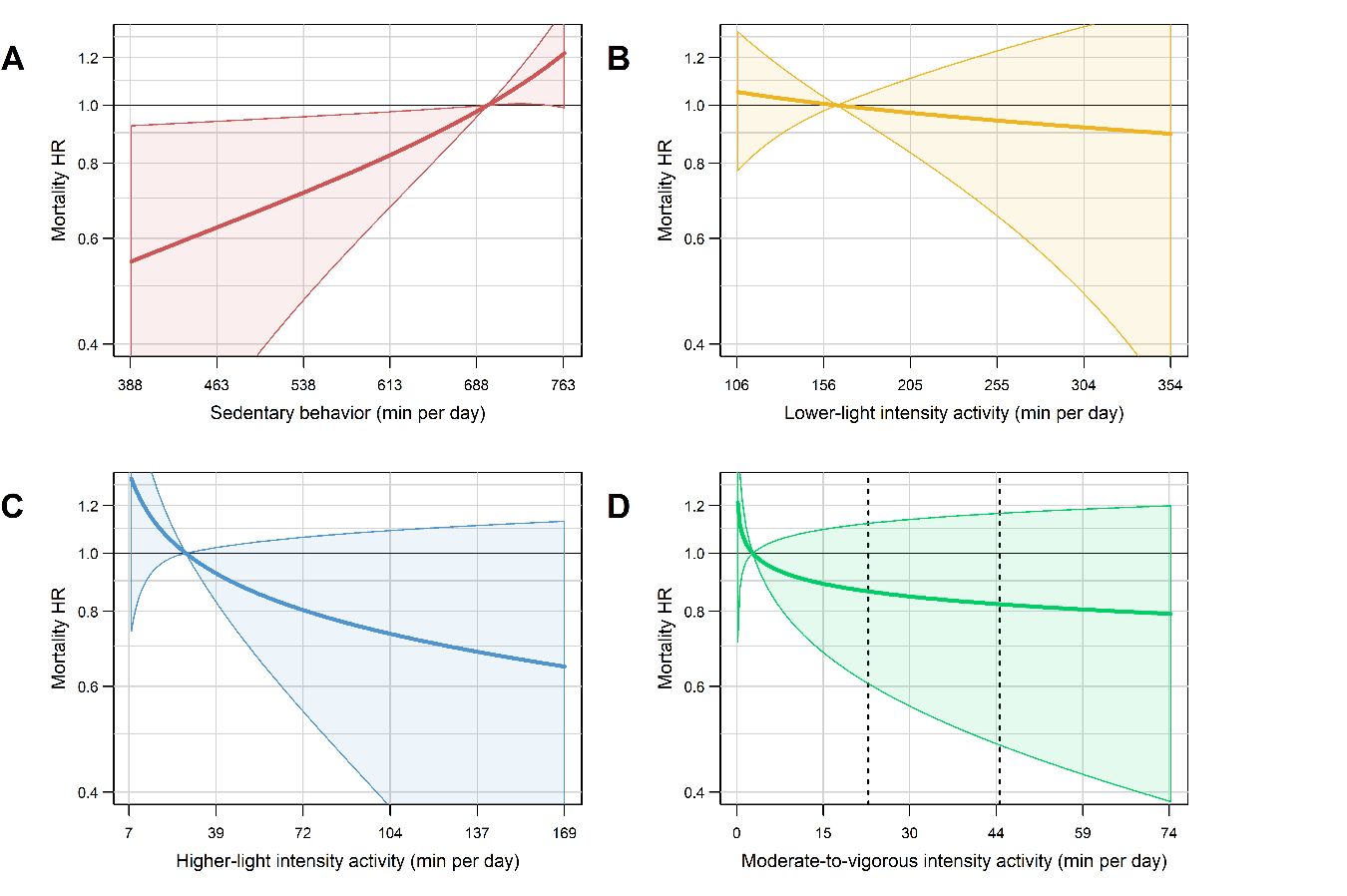


**Figure S1.** Sensitivity analyses in women with >2 years of follow up and without cancer or cardiovascular disease at baseline. Dose-response associations of physical activity of different intensities and sedentary behavior with mortality **(**HRs are compared to the referent composition, the lowest quartile of total activity). Average awake wear time is 14.9 (SD = 1.3) hours per day.

Each line represents time in a behavior while proportionally^a^ reducing the others. Shaded areas represent the 95% confidence intervals.

HR: hazard ratio.

^a^ Proportional to the referent composition, i.e., women in the lowest quartile of total activity: 3 min in moderate-to-vigorous, 27 min higher-light, 162 min lower-light intensity activity, and 701 min in sedentary behavior per day.
